# Inflammatory profiling of patients with familial amyloid polyneuropathy

**DOI:** 10.1186/s12883-019-1369-4

**Published:** 2019-06-28

**Authors:** Estefania P. Azevedo, Anderson B. Guimaraes-Costa, Christianne Bandeira-Melo, Leila Chimelli, Marcia Waddington-Cruz, Elvira M. Saraiva, Fernando L. Palhano, Debora Foguel

**Affiliations:** 1 Instituto de Bioquímica Medica Leopoldo de Meis, Universidade Federal do Rio de Janeiro, Rio de Janeiro, Brazil; 2 Instituto de Microbiologia Paulo de Goes, Universidade Federal do Rio de Janeiro, Rio de Janeiro, Brazil; 3 Instituto de Biofísica Carlos Chagas Filho, Universidade Federal do Rio de Janeiro, Rio de Janeiro, Brazil; 4 Serviço de Anatomia Patológica do Hospital Universitário Clementino Fraga Filho, Universidade Federal do Rio de Janeiro, Rio de Janeiro, Brazil; 5 Serviço de Neurologia do Hospital Universitário Clementino Fraga Filho, Universidade Federal do Rio de Janeiro, Rio de Janeiro, Brazil

**Keywords:** Amyloid, Transthyretin, Biomarkers, Cytokines, Familial amyloid polyneuropathy

## Abstract

**Background:**

Familial amyloid polyneuropathy (FAP) or ATTRv (amyloid TTR variant) amyloidosis is a fatal hereditary disease characterized by the deposition of amyloid fibrils composed of transthyretin (TTR). The current diagnosis of ATTRv relies on genetic identification of TTR mutations and on Congo Red-positive amyloid deposits, which are absent in most ATTRv patients that are asymptomatic or early symptomatic, supporting the need for novel biomarkers to identify patients in earlier disease phases allowing disease control.

**Methods:**

In an effort to search for new markers for ATTRv, our group searched for nine inflammation markers in ATTRv serum from a cohort of 28 Brazilian ATTRv patients.

**Results:**

We found that the levels of six markers were increased (TNF-α, IL-1β, IL-8, IL-33, IFN-β and IL-10), one had decreased levels (IL-12) and two of them were unchanged (IL-6 and cortisol). Interestingly, asymptomatic patients already presented high levels of IL-33, IL-1β and IL-10, suggesting that inflammation may take place before fibril deposition.

**Conclusions:**

Our findings shed light on a new, previously unidentified aspect of ATTRv, which might help define new criteria for disease management, as well as provide additional understanding of ATTRv aggressiveness.

**Electronic supplementary material:**

The online version of this article (10.1186/s12883-019-1369-4) contains supplementary material, which is available to authorized users.

## Background

Familial amyloid polyneuropathy (FAP) or ATTRv (amyloid TTR variant) amyloidosis is an autosomal dominant hereditary disease characterized by the accumulation of amyloid fibrils in peripheral nerves, the gastrointestinal tract and the heart [1, 2]. Disease onset is intimately associated with mutations in the *TTR* gene, located in the chromosome 18, which gives rise to a 55 kDa plasmatic protein named transthyretin [1]. Transthyretin (TTR) is a tetrameric protein expressed and secreted mainly by the liver, but also by the choroid plexus epithelia in the brain [1]. Once in the plasma or in the cerebrospinal fluid, TTR acts as a retinol-binding protein and thyroxine transporter across the body and the brain [1]. More than 100-point mutations in the *TTR* gene have been described worldwide and most of them culminate in the production of dysfunctional TTR with a high thermodynamic instability compared to the wild-type protein [3, 4]. Only a handful of mutations are not pathogenic, such as the T119 M mutation [3, 5]. Most TTR mutations have a high propensity to aggregate under denaturing and even physiological conditions [6], forming amyloid fibrils that deposit in various tissues and organs, causing organ dysfunction [1]. The Val30Met (V30 M) variant is the most common mutation affecting a large population of people worldwide [1, 7]. In particular, a recent study [8] suggested an importance of late-onset cases of V30 M amyloidosis in addition to conventional early-onset studies cases.

The diagnosis of ATTRv is challenging, often relying on genetic tools to identify TTR mutations as well as on the identification of Congo Red-positive amyloid deposits in biopsies usually taken from sural nerve and salivary glands [7, 9]. ATTRv patients have been treated by liver transplantation (LT), since the liver is the major organ of TTR production. Unfortunately, LT presents mortality risks and it is not available to all patients [9]. More recently, a new drug (Tafamidis) that works by stabilizing the TTR protein is available in several countries showing effective results in controlling disease progression [10], but this drug is only available for patients that fulfill certain criteria, such as presenting an amyloid-positive biopsy. However, the amount of ATTRv patients that present ATTRv symptoms without having positive biopsies is significant [11, 12], reinforcing the need for new criteria and novel biomarkers to choose patients that should receive immediate treatment.

Most physicians and pathologists have regarded ATTRv as a disease without an inflammatory component, since most biopsies and ex vivo analysis showed no leukocyte infiltration [13]. Nevertheless, the presence of proinflammatory markers such as TNF-α and IL-1β has been described in biopsies of ATTRv patients [13, 14]. Interestingly, the levels of proinflammatory and oxidative markers in ex vivo tissue positively correlate with the scoring stage proposed by Coutinho and colleagues in ATTRv patients, which is an index used to discriminate disease progression [15]. In addition, an animal model of ATTRv showed increased levels of inflammation-related transcripts in both liver and heart, suggesting that inflammation might play an important role in ATTRv progression [16].

Although, these data indicate a possibility that inflammation may play a role in ATTRv pathogenesis, there are no data available showing which specific inflammatory components are altered in humans. Herein, we searched for inflammation markers in serum collected from a cohort of 28 ATTRv patients in comparison to age and sex-matched controls. We found alterations in six out of nine cytokine levels (TNF-α, IL-1β, IL-8, IL-33, IFN-β, IL-10 and IL-12) compared to healthy age-matched subjects. Interestingly, asymptomatic patients (FAP 0) already presented high levels of some cytokines, suggesting that inflammation may take place before fibril deposition. Notably, in the case of TNF-α, cytokine levels positively correlated with disease progression, indicating a deeper involvement of this cytokine in ATTRv pathogenesis. Our findings shed light to a new, previously unidentified aspect of ATTRv, which might help understanding the aggressiveness of disease and vulnerability of affected individuals.

## Methods

### Human serum

Serum from 28 patients genetically and clinically diagnosed with ATTRv (17 males and 11 females; male/female ratio 1.5:1) were collected from the University Hospital Clementino Fraga Filho of the Federal University of Rio de Janeiro (HUCFF). Serum was collected using standard hospital procedures. Briefly, Vacutainer® tubes were used to collect 5–10 mL of blood. Samples were centrifuged after clotting and readily frozen at − 80 °C. All patients carried the Val30Met mutation. Additionally, serum from 24 healthy donors with a similar female/male ratio was collected and used as control (HS; healthy subjects). We have classified the asymptomatic patients (or asymptomatic carriers) as those patients diagnosed with ATTRv by means of genetic screening, but that do not present ATTRv symptoms yet. This study was approved by the National Committee in Research Ethics (CONEP; CAAE #03102012.4.1001.5257) and informed consent was obtained from all donors. For some of the cytokines measured, the amount of sera available was below the amount necessary for measurement and thus, was excluded from analysis.

### Cytokine quantification

Cytokines were analyzed in whole serum using enzyme-linked immunosorbent assays (ELISA) and commercially available kits: IL-8, IL-33, IL-10, IL-6, IL-12, TNF-α (Peprotech), IL-1β (Invitrogen) and IFN-β and Cortisol (Lifetech Biotechnologies) following the specific manufacturer’s instructions.

### Statistical analysis

All statistical analysis was performed using non-parametric two-tailed Student’s t-test and post-hoc Mann-Whitney test or one-way ANOVA followed by post-hoc Tukey or Kruskal-Wallis test.

## Results

In this study, we included a total of 28 patients previously diagnosed with ATTRv and scored with the scoring system by Coutinho [15] to evaluate disease progression (termed herein FAP 0 = asymptomatic; FAP 1 = unimpaired ambulation; mostly mild sensory, motor, and autonomic neuropathy in the lower limbs; FAP 2 = assistance with ambulation required; mostly moderate impairment progression to the lower limbs, upper limbs, and trunk; FAP 3 = wheelchair-bound or bedridden; severe sensory, motor, and autonomic involvement of all limbs). Twenty-five of them were considered early onset (< 50 years old) and three were considered late-onset (> 50 years old). To better analyze the parameters chosen, the groups were matched to the control group (healthy subjects; HS) and no significant differences in terms of age were observed (*p* > 0.05, One-way ANOVA with Tukey correction, Table 1). In all patients, the diagnosis was confirmed by histopathology of biopsied material, clinical criteria and genetic sequencing of the *TTR* gene being all patients V30 M [7]. With the exception of FAP 0, among our groups of patients, the ratio between male:female had prevalence for males, as noticed in other studies (Table 1) [17]. The patient means ages in FAP 0, 1 and 2–3 sub-groups was 27.5, 38.1 and 35.4, respectively (Table 1).

**Table 1:** Patient’s Characteristics

	Healthy Subjects (HS)	FAP 0	FAP 1	FAP 2-3
Number of subjects	24	6	16	6
Male: Female Ratio	2:1	1:5	2.2:1	5:1
Mean age (range)	36.5 (25-61)	27.5 (24-31)	38.1 (26-71)	35.4 (24-58)

### Comparing the levels of pro- and anti-inflammatory cytokines, interferon-β and cortisol between healthy subjects (HS) and ATTRv patients

**Fig. 1:**
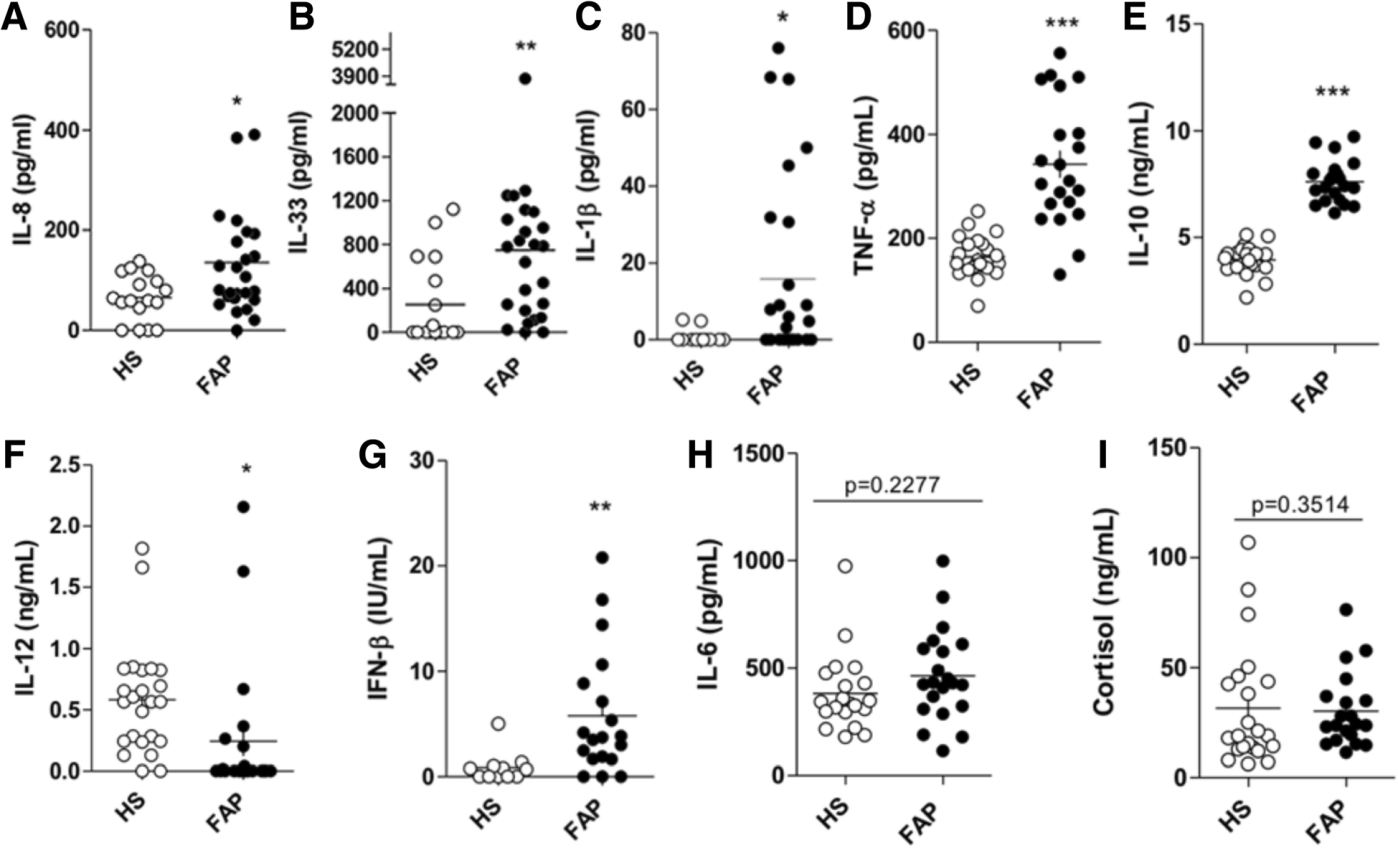
Sera from ATTRv patients exhibit altered immune response. Serum from 28 patients diagnosed positive for ATTRv and 24 age- and sex-matched healthy subjects were collected, and nine different cytokines and immune system associated-molecules were measured: IL-8 (**a**), IL-33 (**b**), IL-1β (**c**), TNF-α (**d**), IL-10 (**e**), IL-12 (**f**), IFN-β (**g**), IL-6 (**h**) and cortisol (**i**). Data were analyzed using unpaired Student’s t test followed by Mann Whitney test. *p < 0.05, **p < 0.01 and ***p < 0.001

Although previous studies have reported elevated levels of pro-inflammatory molecules such as TNF-α and IL-1β in biopsies from ATTRv patients [13, 14, 18], we asked here whether these pro-inflammatory cytokines were also increased in serum of ATTRv patients and whether this increase has any correlation with disease progression. We also assessed five additional cytokines, IL-8, IL-33, IL-10 and IFN-β. Both IL-8 and IL-33 have been associated with promoting inflammation and inducing the recruitment of neutrophils to tissues [19, 20]. Neutrophils are innate immune cells known to be activated by amyloid fibrils in vitro and in vivo [21]. The anti-inflammatory cytokine IL-10 can modulate negatively pro-inflammatory cytokine production by phagocytes and other cells, thus decreasing excessive inflammation [22]. We also evaluated the level of IFN-β in our samples, which is a type I interferon molecule that can modulate microbial and autoimmune responses [23, 24]. In Fig. 1, where all ATTRv patients were grouped together, we observed that ATTRv patients had increased levels of IL-8 (Fig. 1a; 135.5 ± 25.21 pg/mL compared to 65.97 ± 25.14 pg/mL in HS), IL-33 (Fig. 1b; 748.2 ± 195.5 pg/mL compared to 252.1 ± 94.32 pg/mL in HS), IL-1β (Fig. 1c; 15.13 ± 4.659 pg/mL compared to 0.8417 ± 0.5678 pg/mL in HS), TNF-α (Fig. 1d; 342.6 ± 26.09 pg/mL compared to 164.4 ± 8.067 pg/mL in HS), IL-10 (Fig. 1e; 7.609 ± 0.2177 ng/mL compared to 3.970 ± 0.1362 ng/mL in HS), IFN-β (Fig. 1g; 5.770 ± 1.367 IU/mL compared to 0.8739 ± 0.4395 IU/mL in HS). We also evaluated the levels of IL-12, which is mainly produced by phagocytes and can increase the production of IL-8 in neutrophils [25]. Interestingly, we observed a decrease in IL-12 levels (Fig. 1f; 0.2425 ± 0.1203 ng/mL compared to 0.5825 ± 0.09183 ng/mL in HS). We also evaluated IL-6, a cytokine that has dual anti- and pro-inflammatory effects in many different tissues [26], and cortisol, a glucocorticoid that can modulate energy metabolism and suppress immune responses [27] in the serum of ATTRv patients compared to healthy subjects. The levels of both molecules were unchanged when compared to healthy subjects (Fig. 1h, i).

### Asymptomatic patients (FAP 0) already exhibit altered levels of pro- and anti-inflammatory cytokines

**Fig. 2:**
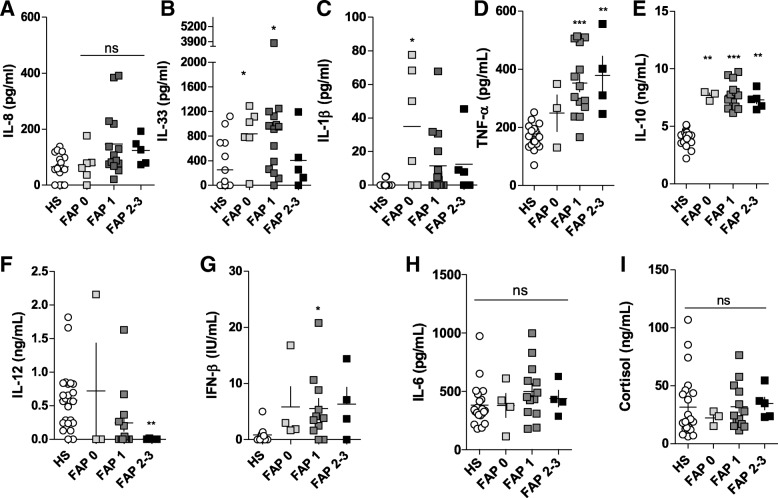
Sera from asymptomatic and symptomatic ATTRv patients exhibit altered immune response. Serum from 28 patients diagnosed positive for ATTRv and 24 age- and sex-matched healthy subjects were collected, and nine different cytokines and immune system associated-molecules were measured: IL-8 (**a**), IL-33 (**b**), IL-1β (**c**), TNF-α (**d**), IL-10 (**e**), IL-12 (**f**), IFN-β (**g**), IL-6 (**h**) and cortisol (**i**). ATTRv patients were scored by Coutinho index (12) and separated by aggressiveness: FAP 0 = asymptomatic; FAP 1 = mild symptoms; FAP 2–3 = moderate to aggressive symptoms. Data were analyzed using unpaired one-way ANOVA followed by Kruskal-Wallis test. *p < 0.05, **p < 0.01 and ***p < 0.001

To evaluate whether the levels of the cytokines here investigated change with disease progression, we plotted their levels in FAP 0, 1 and 2–3 patients and compared to the HS (Fig. 2). We observed that FAP 0 patients already exhibited a significant increase in levels of IL-33, IL-1β and IL-10 (Fig. 2b, c and e), suggesting that the alterations in immune response might occur before amyloid deposition and symptom appearance. In the case of IL-10, the increment already observed in FAP 0 persists with disease progression with no further alteration in other disease stages (Fig. 2e). In the case of IL-33, a similar scenario was observed with the exception that in FAP 2–3 patients this cytokine decreased, presenting similar values as the HS (Fig. 2b). Notably, we found that the levels of TNF-α increased progressively with disease progression, and was significantly different from the HS (Fig. 2d). For all the other cytokines, namely IL-8, IL-12, IL-6, IFN-β and cortisol, their levels did not change and were not significantly different from the HS (Fig. 2a, f-i).

In summary, we presented here a panel of several cytokines from 28 ATTRv patients demonstrating the presence of altered pro- and anti-cytokine levels in serum. These data provide new knowledge regarding ATTRv pathogenesis, suggesting that a relevant immune component exists in ATTRv which opens the possibility for new biomarkers for disease diagnostics (Table 2).

**Table 2:** Summary of cytokines levels in FAP disease stages compared to healthy subjects

	IL-8	IL-6	IL-33	IL-10	IL-1β	TNF-α	IFN-β	IL-12	Cortisol
FAP 0	unchanged	unchanged	↑	↑	↑	unchanged	unchanged	unchanged	unchanged
FAP 1	unchanged	unchanged	↑	↑	unchanged	↑	↑	unchanged	unchanged
FAP 2-3	unchanged	unchanged	unchanged	↑	unchanged	↑	unchanged	↓	unchanged

## Conclusions

The most common mutation leading to ATTRv is V30 M, causing TTR to aggregate forming amyloid fibrils [7]. The majority of patients present polyneuropathy and other complications such as diarrhea/constipation, and sexual impotence among others [1, 7]. However, the mechanism leading to these disorders are unknown. The presence of extracellular deposits of amyloid fibrils composed of wild-type or mutated TTR is shared by all patients [1], but whether these deposits are responsible for disease symptoms is still unclear. For many decades, ATTRv was thought to be a disease in which inflammation was not present [13], due to the lack of leukocyte infiltration observed in ex vivo tissues [13].

Several studies have described the presence of inflammatory markers in ex vivo biopsies [13, 14, 18], suggesting that nerves and surrounding tissue might produce pro- and anti-inflammatory molecules as a stress response or to modulate disease progression. However, no change in leukocyte markers was found, suggesting that the sural nerve or peripheral tissue might be the source of such inflammation [13]. Although the source of the serum TNF-α described by our group is unknown, we cannot discard that inflammatory cells recruited to the sural nerve in response to TTR deposits might be responsible for its production. Another possibility is the expression of TNF-α by Schwann cells present in the sural nerve [28]. Moreover, it is yet unknown whether the inflammatory cytokines detected in this study are primarily related to the pathogenesis or a secondary effect of tissue damage. In addition, many ATTRv patients report neuropathic pain [29], which may modify cytokine profiles [30].

Here, we measured in serum of 28 ATTRv patients the levels of 9 different inflammation-associated molecules and described that ATTRv patients have altered levels of these cytokines, some of them involved in pro- and anti-inflammatory response. In addition, asymptomatic patients already have altered levels of IL-33, IL-1β and IL-10 levels. These altered immune responses observed in asymptomatic patients are a novel finding and suggest that the body is reacting to the disease before any amyloid deposition or tissue degeneration occurs. We couldn’t test sex-specific differences in our cohort due to the small number of male and female patients. However, Kurian and colleagues have observed sex-specific changes in blood cells gene expression from ATTRv patients, suggesting that inflammatory gene markers in circulating blood cells might be influenced by sexual dimorphisms [31]. Curiously, in their study, a correlation of upregulated immune/inflammatory genes was observed among symptomatic versus asymptomatic males, but not in in females [31]. One important limitation to this study is the difficulty to recruit patients in Brazil for serology studies, thus it remains unknown whether these cytokine changes in serum would also be present in patients with a different TTR mutation and a non-TTR neuropathy (Chronic inflammatory demyelinating polyneuropathy; CIDP).

One possibility is that the synthesis and folding process of the mutated and unstable TTR in the liver requires more energy and thus, may cause endoplasmic reticulum (ER) stress and the activation of the liver unfolding protein response (UPR). ER stress and the activation of UPR in liver were shown to cause pro-inflammatory cytokines production [32, 33], which could fuel inflammation by further activating liver macrophages (Kupffer cells) as seen in other non-amyloid disease [34, 35]. In a recent publication by Kurian and collaborators, it was shown that genes related to eIF2 pathway were downregulated in all ATTRv patients in relation to the asymptomatic ones. This pathway is one arm of the protein unfolding response and its activation could be related to initiation of the proinflammatory response in ATTRV patients described here [31]. Interestingly, Buxbaum and colleagues showed in a mouse model of ATTRv that liver from mice that presented no amyloid deposits exhibited altered expression of diverse transcripts associated with innate immunity and inflammation [16]. Our data and the one from that study suggest that before amyloid deposition occurs, gene expression and protein levels of inflammatory markers are altered in mice [16] and humans with ATTRv. It remains unknown whether this might be a way to modulate disease progression or is merely a consequence of misfolded TTR local synthesis. A better understanding of why these alterations occur in serum and whether the liver plays an important role in this phenomenon may provide new insights to improve the quality of life of patients that also undergo domino liver transplant. In this procedure, a liver failure patient receives a liver from an ATTRv patient. However, a five-year study described that 35% of patients that underwent domino liver transplantation presented ATTRv amyloidosis earlier than donor ATTRv patients [36]. These data indicate that ATTRv patients may have altered liver capacity, putting domino liver recipients at risk.

Another possibility is that the inflammatory response is a reaction to circulating small oligomers. Amyloid oligomers are formed before fibril deposition, are toxic to cells [37], and elicit inflammation when presented to immune cells [21]. Small, toxic oligomers can also be produced in situ after the cleavage of mature fibrils through the action of local proteases [21].

Altered immune response and inflammation can affect a myriad of pathways including glucose [38], and lipid metabolisms [39], among others. The patients with ATTRv also present gastrointestinal symptoms, cachexia, malnutrition, diarrhea and others [1, 7]. Cytokines such as IL-1β are important players in inflammation-induced anorexia and weight loss, due to its action in the hypothalamus-pituitary-adrenal axis (HPA) [40]. One hypothesis is that altered IL-1β levels might change neuroendocrine pathways leading to anorexia and thus, cachexia in ATTRv patients. IL-1β also is known as a modulator of other pro-inflammatory cytokines [41] that might also influence neuroendocrine metabolism. Interestingly, in some patients, cachexia is not correlated with dysautonomia and malabsorption.

The novel observation of alterations in immune response in serum from ATTRv patients suggests an important role of inflammation in ATTRv pathogenesis. This new focus on inflammation in ATTRv pathogenesis and progression provides new directions towards understanding this fatal disease and improving clinical criteria for choosing ATTRv patients that should receive Tafamidis treatment. The drug has already showed positive results in clinical trials [10], but due to current clinical criteria is being administered to ATTRv patients that are already debilitated by disease. If these inflammatory markers could be evaluated in other ATTRv populations showing a similar profile with disease progression, we envision the possibility of using them as ATTRv biomarkers, which could be assessed vary rapidly and very easily.

## Additional file

Additional file 1: Raw Data from Patients. (XLSX 12 kb)

## Data Availability

Raw data were submitted as Additional file 1.

## Abbreviations

ATTRv: Amyloid transthyretin variant
FAP: Familial amyloidotic polyneuropathy
HS: Healthy subjects
LT: Liver transplantation
UPR: Unfolding protein response
